# A randomized controlled phase IIb wound healing trial of cutaneous leishmaniasis ulcers with 0.045% pharmaceutical chlorite (DAC N-055) with and without bipolar high frequency electro-cauterization versus intralesional antimony in Afghanistan

**DOI:** 10.1186/s12879-014-0619-8

**Published:** 2014-11-25

**Authors:** Hans-Christian Stahl, Faridullah Ahmadi, Ulrike Schleicher, Rainer Sauerborn, Justo Lorenzo Bermejo, Mohammed Latif Amirih, Ibrahim Sakhayee, Christian Bogdan, Kurt-Wilhelm Stahl

**Affiliations:** Institute of Public Health, University Hospital Heidelberg, Heidelberg, Germany; Provincial Balkh Hospital, Mazar-e-Sharif, Afghanistan; Mikrobiologisches Institut – Klinische Mikrobiologie, Immunologie und Hygiene, Friedrich-Alexander-Universität (FAU) Erlangen-Nürnberg und Universitätsklinikum Erlangen, Erlangen, Germany; Institut für Medizinische Biometrie, Universität Heidelberg, Heidelberg, Germany; Waisenmedizin e.V. – PACEM, Freiburg, Germany

**Keywords:** Cutaneous leishmaniasis, Wound healing, DAC N-055, Sodium stibogluconate, High-frequency bipolar electrocauterization

## Abstract

**Background:**

A previously published proof of principle phase IIa trial with 113 patients from Kabul showed that bipolar high-frequency (HF) electro-cauterization (EC) of cutaneous leishmaniasis (CL) ulcers and subsequent moist wound treatment (MWT) closed 85% of all *Leishmania (L.) tropica* lesions within 60 days.

**Methods:**

A three-armed phase IIb, randomized and controlled clinical trial was performed in Mazar-e-Sharif. *L. tropica*- or *L. major*-infected CL patients received intradermal sodium stibogluconate (SSG) (Group I); HF-EC followed by MWT with 0.045% DAC N-055 (Group II); or MWT with 0.045% DAC N-055 in basic crème alone (Group III). The primary outcome was complete epithelialisation before day 75 after treatment start.

**Results:**

87 patients enrolled in the trial were randomized into group I (n = 24), II (n = 32) and III (n = 31). The per-protocol analysis of 69 (79%) patients revealed complete epithelialisation before day 75 in 15 (of 23; 65%) patients of Group I, in 23 (of 23; 100%) patients of Group II, and in 20 (of 23; 87%) patients of Group III (p = 0.004, Fisher’s Exact Test). In the per-protocol analysis, wound closure times were significantly different between all regimens in a pair-wise comparison (p = 0.000039, Log-Rank (Mantel-Cox) test). In the intention-to-treat analysis wound survival times in Group II were significantly different from those in Group I (p = 0.000040, Log-Rank (Mantel-Cox) test). Re-ulcerations occurred in four (17%), three (13%) and seven (30%) patients of Group I, II or III, respectively (p = 0.312, Pearson Chi-Square Test).

**Conclusions:**

Treatment of CL ulcers with bipolar HF-EC followed by MWT with 0.045% DAC N-055 or with DAC N-055 alone showed shorter wound closure times than with the standard SSG therapy. The results merit further exploration in larger trials in the light of our current knowledge of *in vitro* and *in vivo* activities of chlorite. Clinicaltrials.gov ID: NCT00996463. Registered: 15^th^ October 2009.

**Electronic supplementary material:**

The online version of this article (doi:10.1186/s12879-014-0619-8) contains supplementary material, which is available to authorized users.

## Background

In developed countries chronic wounds are an advanced age disease. In Afghanistan, cutaneous leishmaniasis (CL) ulcers resulting from parasite infections disfigure non-covered parts of the body especially at younger age. Advanced age wounds elicit industrial R&D efforts, whereas particularly in the Near, Middle and Far East CL wounds constitute a neglected field of clinical research, although their incidence of approximately 0.5% to 1% is of the same magnitude [1],[2].

In 1986, a randomized controlled trial (RCT) has shown that moist dressing with a sodium chlorite (NaClO_2_)-based drug is beneficial for rapid wound cleansing and granulation [3]. If not further concentrated under vacuum (which is commonly practiced with industrial NaClO_2_), pharmaceutical chlorite contains a chlorine peroxide contaminant, formerly called tetradecachlorooxygen (TCDO), a chemical name refuted by the German Health Authorities [4]. The chlorine peroxide seems to be important for systemic regenerative effects in stem cell compartments of rats [5],[6]. Recent advances in bacterial heme protein biochemistry [7],[8] have reformed our understanding of chlorite biochemistry [9] showing that *in vivo* reactions of chlorite with heme analogues can either produce hypochlorite, which in turn can react with H_2_O_2_ to form singlet oxygen ^1^O_2_, or which can dismutate to Cl^-^ and ^1^O_2_. In animal experiments with non-thermal (NTP) [10] or cold atmospheric plasma (CAP) [11] lower μM levels ^1^O_2_ have shown to induce wound healing.

Simple physical wound debridement practised with bi-polar high frequency electrosurgical cauterisation (HF-EC) as a first step seemed to be of crucial importance [12] to achieve faster wound healing than obtained with sodium stibogluconate (SSG) [13]. As recently advocated [14], special attention should be given to the wound disease character of Old World Cutaneous Leishmaniasis (OWCL) lesions with frequent bacterial and fungal contaminations [15] which are typical for chronic wounds [16].

Within the present randomized controlled three-armed clinical trial we investigated the benefit of applying local wound treatment to OWCL ulcers. The overall aim of this RCT phase IIb trial in Mazar-e-Sharif, Afghanistan, was (a) to confirm previous results [12] using bipolar HF-EC combined with subsequent moist wound treatment (MWT) with 0.045 % of the pharmaceutical sodium chlorite solution (DAC N-055); and to directly compare the results (b) with topical anti-parasitic SSG and (c) with 0.045% DAC N-055 MWT alone, known to promote tissue regeneration [3],[5],[6]. The trial was further encouraged by the previous successful treatment of four patients with facial lupoid leishmaniasis, in whom the topical application of 0.045% DAC N-055 led to a rejuvenation of the faces [17],[18].

## Methods

### Trial design

The study was designed as a mono-centric, three armed, open label, randomized (1:1:1), controlled, phase IIb trial with tissue biopsy [19] without any amendments to the protocol after trial start.

### Ethics

Ethical clearance was obtained from the Ethics Committees of the Medical Faculties of Heidelberg and Erlangen in Germany and the International Review Board at the Ministry of Public Health in Kabul, Afghanistan (Clinicaltrials.gov ID: NCT00996463. Registered: 15^th^ October 2009). As nearly all patients could neither read nor write or count, an oral informed consent before patient screening was obtained by the medical doctor after thorough and comprehensible explanation of the aims and the protocol of the clinical trial.

### Participants

Patients presenting CL lesions with *Leishmania*-positive Giemsa smears without prior CL treatment were included. Exclusion criteria were: age <12 years, more than one skin lesion (to exclude intra-individual variations in this phase IIb analysis), lesion age >3 months, lesions located on eye lids, lips or nose, drug addiction, co-infection with *Mycobacterium tuberculosis* or HIV, and diabetes. All patients who had agreed to participate in this trial had respected our call regarding patients’ age, lesion age and lesion location. No patients were lost during the screening process due to drug addiction, tuberculosis, HIV infection, or diabetes. Patients not available for follow-up were also ineligible. Medical services and drugs were free of charge and patients received no remuneration.

### Location

The trial was carried out at the Leishmania and Malaria Centre (LMC) of the Provincial Balkh Civil Hospital Mazar-e-Sharif treating 4,000 new CL cases every year [20]. Before trial start the centre was renovated under the supervision of the NGO Waisenmedizin e.V. and equipped with a solar power system guarantying electrical power supply for 24 h per day.

### Data capture system

Cameras, computers, and the internet-based on- and offline electronic case report system Leishmedoc (Waisenmedizin – PACEM e.V. Freiburg, Germany) in combination with Skype™ (Microsoft Corporation, Redmond, USA) communication enabled real-time trial monitoring from Germany.

### Protocol of visits

Patients were registered after informed consent (see Ethics) with demographic details, cell phone numbers and a patient’s identification number on a patient card. After physical examination, the location and initial state of the lesion were documented and referenced by the patient’s identification number and a scale. Six visits were scheduled in the first week, two visits per week from weeks 2 to 4, and one visit per week thereafter until complete wound closure. Follow-up visits were required once a month until day 180 after treatment start.

### Drugs for interventional therapy

Sodium stibogluconate (3 g/30 ml) was imported from India (Albert David Ltd., India). 4.5% (500 mM) alkaline sodium chlorite solution for pharmaceutical use (DAC N-055; Kyrochem GmbH, Wedemark, Germany) was diluted in the constituent formulas of the poly-acrylate jelly and basic crème (Additional files 1 and 2). The German DAC N-055 contains peroxides [21] (Na_2_Cl_2_O_6_ and NaCClO_6_) at an app. 1:10 molar ratio, if produced from chlorine dioxide in a way to minimize the chlorate content. DAC N-055, formerly known as TCDO, promotes tissue regeneration, as does hydrogen peroxide at concentrations <10^-5^ M [22],[23]. In contrast to hydrogen peroxide, DAC N-055 is catalase (EC 1.11.1.6) resistant [24]. Vials of sterile distilled water and 1% lidocaïne were purchased from local pharmacies in Mazar-e-Sharif. Jellies were freshly prepared every day by the dermatologist (IS) of the Balkh Province Civil Hospital of Mazar-e-Sharif, who had been trained in the Unguator® Technology (GAKO® International GmbH, Munich, Germany) at the Pharmaceutical Institute of the University Freiburg, Germany. EuRho® DAC 2003 cream (Euro OTC Pharma GmbH, Bönen, Germany) is a magistral preparation available in German pharmacies (Additional file 2).

### Other drugs

**Figure 1:**
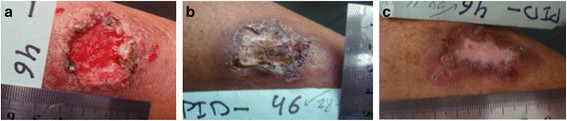
**Photo-documentation of wound epithelialization of crusted lesions after removal of the crusts. (Panel a)** Lesion status prior to the start of the intradermal SSG treatment start at day 0; **(Panel b)** Lesion status after 12 intradermal SSG injections on day 51; **(Panel c)** Lesion status after gentle sloughing off with Prontosan® detergent on day 90. Crusts, that were close to slough off, were removed after 30 min incubation with aseptic 0.1% Prontosan® containing the detergent undecylenamidopropyl betaine. Afterwards, the wound was photo-documented including the patient's identification number and a linear scale to analyse the wound size.

In case of clinically diagnosed wound infections, topical wound disinfection was allowed for 5 consecutive days with saline containing 970 ppm chlorine dioxide (freshly prepared by acidification of 0.27 % DAC N-055 in physiological saline to pH 5). In case of failure, a systemic therapy with antibiotics or with anti-mycotics of little anti-parasitic effect was recommended. Prontosan® (B. Braun Medical AG, Sempach, Switzerland) with the detergent undecylenamidopropyl betaïne (CAS 133798-12-6) at a concentration of 0.1 % was used for gently sloughing off crusts after SSG injections to detect complete epithelialisation (Figure 1).

### Medical devices for interventional therapy

The electrosurgical Minicutter™ with a specially designed bipolar angled forceps with a 1 mM distance holder and a maximum bipolar current mode of 70 mA (HMC 80 HF Chirurgie, KLS Martin, Umkirch, Germany) was imported from Germany.

### Randomization

The LMC principal dermatologist investigator (IS) determined the patients’ eligibility. Each patient was randomly assigned to one of the three regimens by the random allocation generator in the computer-based Leishmedoc system.

### Treatment protocols

The patients’ OWCL lesion was treated (1) by intradermal injections of 0.6 ml SSG according to a protocol used by Zeglin (2009) [25] (Group I), or (2) by aseptic MWT with 0.045% DAC N-055 following a single initial superficial wound debridement with HF-EC which was performed under local anaesthesia after wound cleansing and disinfection with gauzes soaked in physiological saline solution containing 320 ppm chlorine dioxide (pH 5.5 acidified 0.09% DAC N-055) for 15 minutes (Group II), or (3) by MWT with 0.045% DAC N-055 alone (Group III).

The topical treatment schedule was identical in all three regimens: daily treatments (with the exception of Fridays) during the first week, followed by topical treatments at the LMC twice a week until the end of week 4 and thereafter once a week until wound closure. In Group I, the SSG treatment was discontinued after week 4. In Groups II and III, patients dressed their lesion themselves after week 4 with the topical NaClO_2_-basic-crème between their visits at the LMC until lesion closure.

The HF-EC debridement started with a superficial coagulation of the epidermis using the strongest relative current of the Minicutter™ (position 10) for approximately two seconds necessary to boil off the excess physiological saline on the lesion. After the coagulated epidermis was mechanically removed with moist gauze, the parasite infected dermal layer became visible as a reddish granulomatous area and was specifically targeted by a second coagulation until the area turned into a slightly brownish colour. This procedure was performed only once. The wounds of Groups II and III were dressed with a EuRho® DAC 2003 cream preparation (Additional file 2) until lesion closure, with the exception of Group II, in which for 6 days after HF-EC debridement of the CL lesions the wounds were daily dressed with freshly prepared poly-acrylate jelly containing 0.045% DAC N-055 (Additional file 1).

During week 1, patients of Groups II and III and their relatives were trained to dress wounds, receiving 10 g EuRho® DAC 2003 cream containing 0.045% DAC N-055, in a sterile syringe, which allowed self-treatment for 3 weeks.

### Parasitological analyses and *Leishmania*species determination

At the LMC, *Leishmania* parasites within the lesion were confirmed with the slit-skin method described by Al Hucheimi [26]. In addition, skin biopsies were taken from the margins of the lesions and placed into modified Schneider's *Drosophila* insect medium, transported with the German Army to the Microbiology Institute in Erlangen, Germany, where mini-exon polymerase chain reaction (PCR) and multiple restriction fragment length polymorphism analyses for parasite species determination and limiting dilution analyses for determination of the parasite loads per gram biopsy tissue were carried out as previously described [12].

### Outcome

The primary outcome of the study was the ratio of closed versus open wounds at day 75 (D75) in the PP analysis for each regimen.

### Sample size

The specific hypotheses were (i) that the proportion of primary closed lesions before D75 is significantly higher in patients treated with HF-EC with subsequent MWT using 0.045% DAC N-055 (Group II) than in patients who received topical intradermal SSG (Group I); and (ii) that MWT with 0.045% DAC N-055 alone, which is known to exert tissue regenerative activity in vivo [3],[18], also promotes the closure of chronic *Leishmania* lesions (Group III).

The sample size calculation was based on the per-protocol (PP) analysis, defined as all patients evaluable with respect to the primary endpoint. Based on the Reithinger [13] trial and our previous findings in Kabul [12] we defined D75 as endpoint to evaluate a clinically relevant difference of 40% between the percentage of wound closures in Group I (50% at D75) and Group II (90% at D75), respectively. We assumed that SSG chemotherapy does not directly accelerate the wound healing process. In Group III a percentage of 75% at D75 was estimated, since MWT promotes wound healing. A power calculation based on Group I and II showed that 42 patients were needed in each arm to reject the null hypothesis of equal cure rates with a 90% probability using the Fisher’s exact test (Type I error probability 1%). An interim analysis was planned when 50% of the patients (42/2 = 21) had efficacy assessments. Bonferroni adjustment was used to calculate p_1_ = 2p_min_, where p_min_ was the smallest of p_Group I__vs Group II_ versus p_Group I vs Group III_ [27]. Based on the pre-specified stopping boundaries α_1_ = 0.01, β_1_ = 0.15 and α_2_ = 0.1871 and decision rules (Additional file 3), the trial could be stopped at stage I for efficacy.

### Statistical analysis

The present trial is a phase IIb efficacy assessment trial. The intention-to-treat analysis (ITT) is solely added as additional information. Patients that could not be evaluated were patients that were lost immediately after registration before treatment started. Within the group of evaluable patients we distinguished between patients whose lesion could be evaluated with respect to D75 and those that were lost to follow-up before D75 with no wound closure. The former were included in the per-protocol-analysis (PP) and the latter were additionally included in the ITT. Screened patients lost immediately after registration, were not included in our definition of the ITT analysis.

Baseline characteristics were analysed using the Pearson Chi-Square Test or the Kruskal-Wallis Test. Primary outcome was evaluated using the Fisher’s Exact Test. Wound closure times were calculated with Kaplan-Meier survival analysis. Hazard ratios and potential covariates were analysed with Cox-Regression. Re-ulceration rates were compared using the Pearson Chi-Square Test. Statistical analyses were performed with IBM® SPSS® Statistics version 21 (IBM Deutschland GmbH, Ehningen, Germany), except the statistics on the *Leishmania* load per gram biopsy tissue that were calculated with GraphPad Prism version 4.0 (Graphpad Software Inc., La Jolla, CA, USA).

## Results

### Enrolled patients

**Figure 2:**
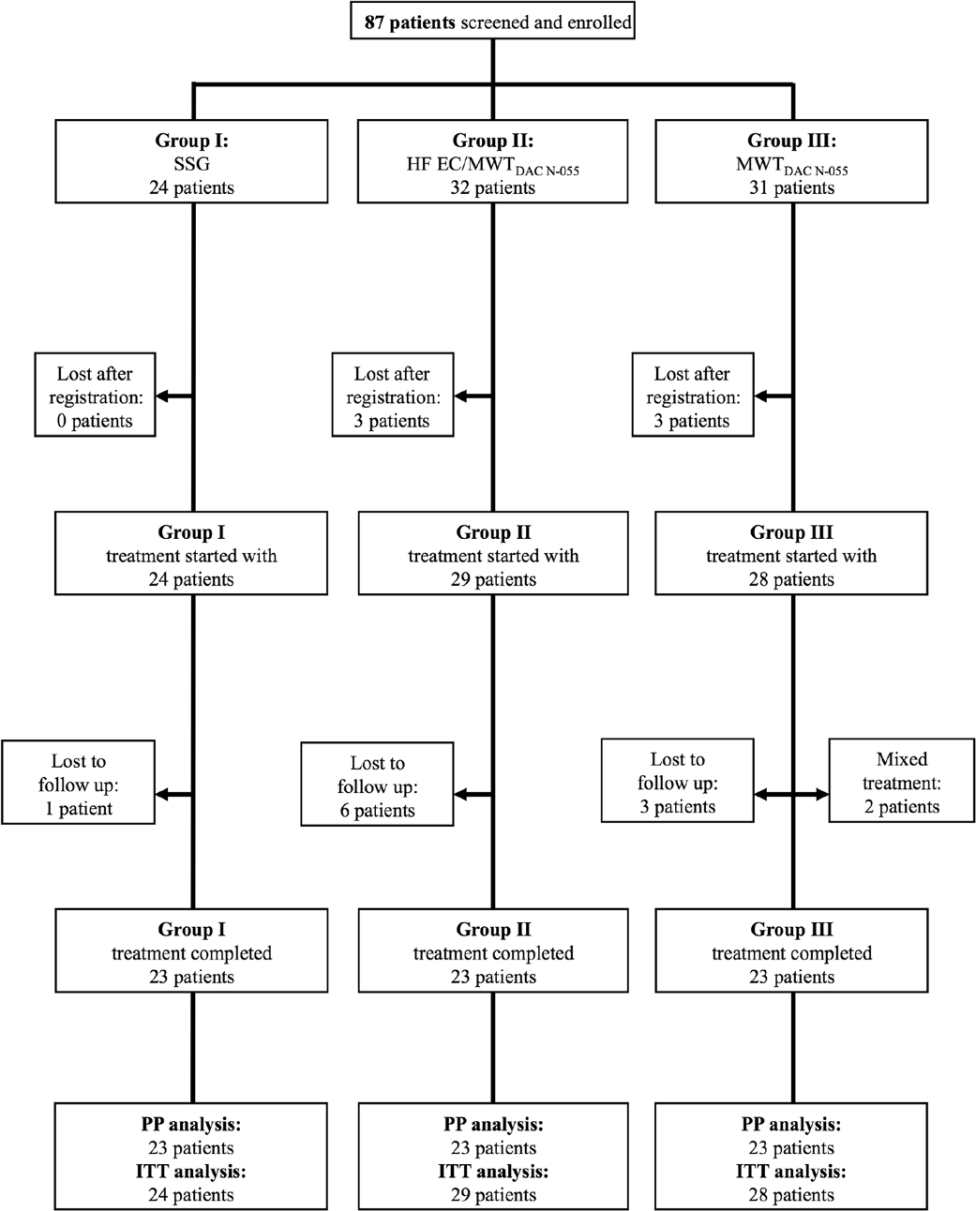
**Flow chart summarizing the enrolment, randomization, and follow-up of study patients.**

In total, 87 patients were enrolled, with 24 (27.5%), 32 (36.8%), and 31 (35.6%) in Groups I, II, and III, respectively (Figure 2). 81 out of 87 patients (93.1%) were suitable for the ITT and 69 (79.3%) for the PP analysis (Additional file 4).

### Recruitment

Patients were enrolled from November 2009 to August 2010. The efficacy value of p_1_ was found smaller than the stopping boundary of α_1_ = 0.01 pre-defined in the protocol. Therefore the study was stopped (Additional file 3).

### Baseline data

Sixty-nine patients (37 [54%] females, 32 [46%] males) with a mean age of 29 years (CI 25-33) followed the protocol. The analysed baseline data were comparable in all three regimens in the PP (Table 1) as well as ITT analysis (Table 2). In 44 out of 69 patients analysed, 28 (64%) lesions were infected with *L. tropica* and 16 (36%) with *L. major. L. major* infections dominated from September to March, *L. tropica* from March to August.

**Table 1:** **Baseline characteristics of the PP patients enrolled in the three treatment groups of the trial**

	Per protocol (PP) evaluation					Test
	Group I	Group II	Group III	Total	p-value	
Male	13	8	11	32		Pearson Chi-Square
Female	10	15	12	37	0.372*	Test (Exact-Sig.)
Age (95% CI)	26 (19-34)	28 (22-33)	33 (25-40)	29 (25-33)	0.308*	Kruskal-Wallis Test
Lesion age (95% CI)	9 (7-11)	8 (7-10)	7 (6-9)	8 (7-9)	0.437*	Kruskal-Wallis Test
Lesion size (95% CI)	3.7 (2.3-5.0)	2.5 (1.7-3.3)	3.0 (2.0-4.1)	3.1 (2.5-3.7)	0.421*	Kruskal-Wallis Test
*Parasite species*						
L. major	4	5	7	16		
L. tropica	5	13	10	28		Pearson Chi-Square
Not determined	14	5	6	25	0.664*	Test (Exact-Sig.)
*Lesion location*						
Head	5	3	2	10		
Trunc	0	0	1	1		
Upper extr.	12	16	14	42		Pearson Chi-Square
Lower extr.	6	4	6	16	0.666*	Test (Exact-Sig.)
*Parasite load according to Giemsa staining*						
Low (+)	12	11	8	31		
Moderate (++)	8	12	14	34		
High (+++)	1	0	1	2		Pearson Chi-Square
Not determined	2	0	0	2	0.541*	Test (Exact-Sig.)
*Biopsy prior to treatment*						
Evaluable	16	18	17	51		Pearson Chi-Square
Not evaluable	7	5	6	18	0.940*	Test (Exact-Sig.)
Parasite load/g tissue (SEM)	2.944 (1.645) × 10^6^	3.081 (1.980) × 10^6^	2.773 (1.785) × 10^6^	2.935 (1.032) × 10^6^	0.636*	Kruskal-Wallis Test

Statistical tests were used as indicated (p < 0.05 indicates a significant proportion, *p-values refer to observations with complete information).

**Table 2:** **Baseline characteristics of the ITT patients enrolled in the three treatment groups of the trial**

	Intention to treat (ITT) evaluation					Test
	Group I	Group II	Group III	Total	p-value	
Male	14	12	15	41		Pearson Chi-Square
Female	10	17	13	40	0.446*	Test (Exact-Sig.)
Age (95% CI)	26 (19-33)	28 (23-33)	32 (25-38)	29 (25-32)	0.252*	Kruskal-Wallis Test
Lesion age (95% CI)	9 (7-10)	9 (8-11)	7 (6-8)	8 (7-9)	0.187*	Kruskal-Wallis Test
Lesion size (95% CI)	3.6 (2.3-4.9)	2.3 (1.7-3.0)	2.7 (1.8-3.6)	2.8 (2.3-3.4)	0.204*	Kruskal-Wallis Test
*Parasite species*						
L. major	5	7	9	21		
L. tropica	5	14	11	30		Pearson Chi-Square
Not determined	14	8	8	30	0.614*	Test (Exact-Sig.)
*Lesion location*						
Head	5	4	4	13		
Trunc	0	0	1	1		
Upper extr.	12	19	16	47		Pearson Chi-Square
Lower extr.	7	5	7	19	0.799*	Test (Exact-Sig.)
*Parasite load according to Giemsa staining*						
Low (+)	13	16	11	40		
Moderate (++)	8	13	16	37		
High (+++)	1	0	1	2		Pearson Chi-Square
Not determined	2	0	0	2	0.534*	Test (Exact-Sig.)
*Biopsy prior to treatment*						
Evaluable	17	23	22	62		Pearson Chi-Square
Not evaluable	7	6	6	19	0.800*	Test (Exact-Sig.)
Parasite load/g tissue (SEM)	2.773 (1.554)x10^6^	3.430 (1.736)x10^6^	2.368 (1.526)x10^6^	2.889 (0.932)x10^6^	0.691*	Kruskal-Wallis Test

Statistical tests were used as indicated (p < 0.05 indicates a significant proportion, * p-values refer to observations with complete information).

### Primary outcome

15 out of 23 (65%), 23 out of 23 (100%), and 20 out of 23 (87%) patients attained complete epithelialisation until D75 in Groups I, II and III, respectively (Group I versus II: p = 0.004 [PP]; Table 3). For the intention to treat results see Table 4.

**Table 3:** **Per protocol analysis of the primary endpoint**

	Per protocol (PP) evaluation				
	Group I	Group II	Group III	Total	p-value
**< D75**	15	23	20	58	
**≥ D75**	8	0	3	11	0.004*
**Unknown**	NA	NA	NA	NA	
**Total**	23	23	23	69	

*Pearson Chi-Square Test (Exact-Sign).

**Table 4:** **Intention to treat analysis of the primary endpoint**

	Intention to treat (ITT) evaluation				
	Group I	Group II	Group III	Total	p-value
**< D75**	15	23	20	58	
**≥ D75**	8	0	4	12	
**Unknown**	1	6	4	11	0.009*
**Total**	24	29	28	81	

*Pearson Chi-Square Test (Exact-Sign).

### Secondary outcome

**Figure 3:**
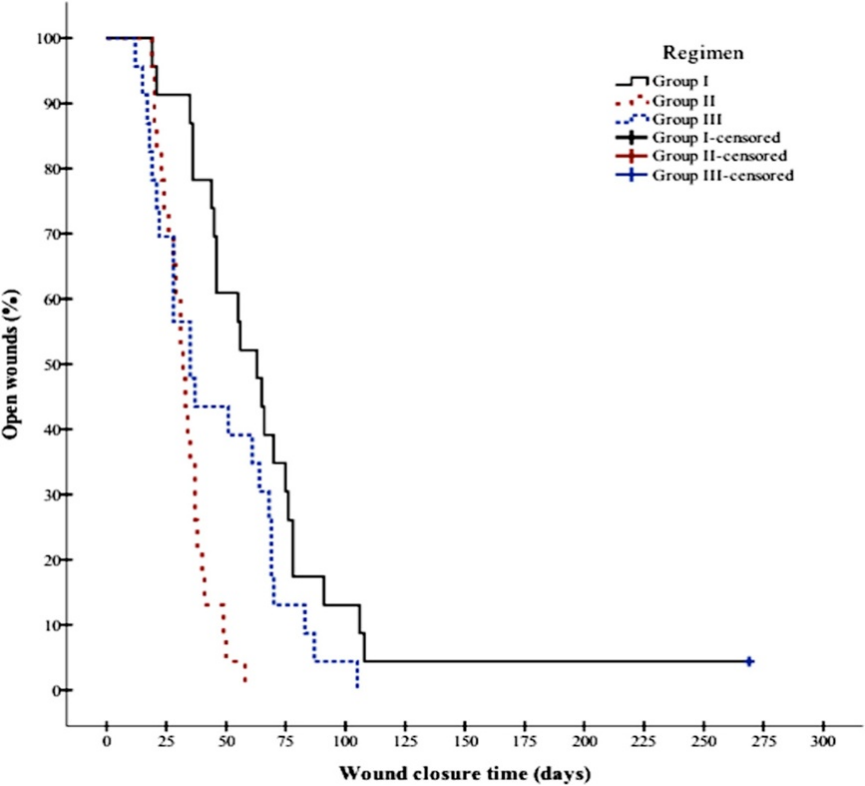
**Wound closure time in PP patients of Group I versus Group II versus Group III.** Statistically significant difference between Group II versus Group I (p = 0.000001, Log-Rank [Mantel-Cox] test). Statistical significant differences were found between all groups (see Table 6).

**Figure 4:**
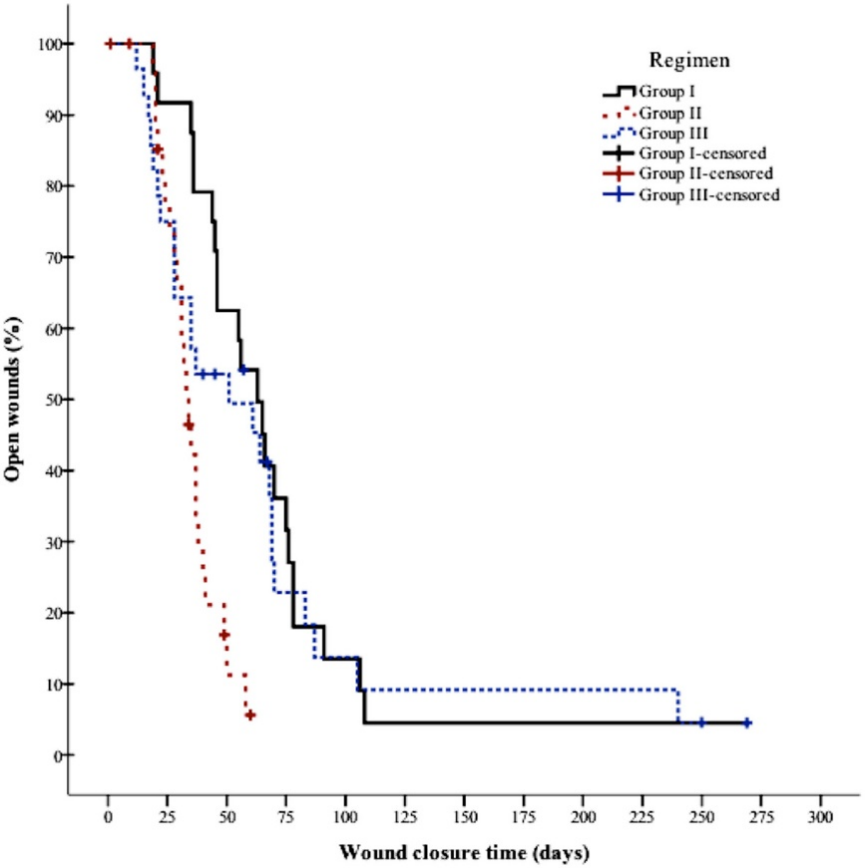
**Wound closure time in ITT patients of Group I versus Group II versus Group III.** Statistically significant difference between Group II versus Group I (p = 0.00004, Log-Rank [Mantel-Cox] test).

The frequent attendance of the study participants allowed plotting wound survival curves (Figures 3 and 4). In a Kaplan-Meier analysis (days until primary wound closure), the survival curves of the three investigated treatments differed significantly in an overall comparison (p = 0.000039 [PP]; p = 0.000616 [ITT], Log-Rank [Mantel-Cox] test). In the ITT analyses, mean and median lesion wound survival times in Group II (35 [CI 30-40]/34 days [CI 29-39]) were significantly different from those in Group I (69 days [CI 50-90]/63 days [CI 50-75]) (Tables 5 and 6); in contrast, the pair-wise comparison of the Group III versus Group I survival times did not reveal significant differences (p = 0.508, Log-Rank [Mantel-Cox] test) (Tables 5 and 6). In the PP analysis, pair-wise comparison for all three groups showed significant differences in wound survival time (p = 0.023 [Group II vs. III]; p = 0.047 [Group III vs. I], Log-Rank [Mantel-Cox] test) (Tables 6 and 7). Hazard ratios of Group II versus Group I equaled to 4.415 (CI 2.219-8.783) in the PP (p = 0.000023) and 3.270 (CI 1.687-6.341) in the ITT analysis (p = 0.000453). The lesion closed three to four times faster in Group II than in Group I.

**Table 5:** **Intention to treat analysis of mean and median wound survival time (days)**

	Intention to treat (ITT) evaluation			
	Group I	Group II	Group III	Overall
**Mean (95% CI)**	69 (50-90)	35 (30-40)	66 (41-91)	58 (46-70)
**Median (95% CI)**	63 (50-75)	34 (29-39)	51 (17-85)	44 (32-56)

**Table 6:** **P-values: pairwise comparison of the survival functions**

	Group I	Group II	Group III
**Group I**		0.000001/0.000040	0.047032/0.507623
**Group II**	0.000001/0.000040		0.022813/0.013589
**Group III**	0.047032/0.507623	0.022813/0.013589	

Log-Rank (Mantel-Cox) test, p-values refer to the per-protocol (left value) and the intention-to-treat analysis (right value).

**Table 7:** **Per protocol analysis of mean and median wound survival time (days)**

	Per protocol (PP) evaluation			
	Group I	Group II	Group III	Overall
**Mean (95% CI)**	69 (49-89)	33 (29-37)	45 (34-56)	49 (41-57)
**Median (95% CI)**	63 (47-79)	32 (27-37)	35 (21-49)	37 (31-43)

### Covariates analysis

In the Cox-regression analysis, gender, age of the patient age, and age of the lesion were no significant covariates for primary closure. Lesion size at baseline was a significant (p = 0.016) covariate with a hazard ratio of 0.883 (CI 0.798-0.977) in the PP, but not in the ITT analysis (p = 0.312) with a hazard ratio of 0.954 (CI 0.872-1.045).

### Non-desired effects (NDEs)

In the PP analysis, re-ulceration rates were similar in Group I (four patients) and in Group II (three patients), but were more frequent in Group III (seven patients) (p = 0.312, Pearson Chi-Square test). No final closure was documented for two patients (D131 and D269), one patient (D186), and five patients (D90, D142, D143, D143, D176) in Groups I, II, and III respectively. The number of patients in the three arms of this trial was to small to make a meaningful comparison as to which treatment mode yields the best cosmetic scar outcome. However, in Group II, the flat scars showed the typical livid borders with hypo-pigmented central zones, taking weeks to months to fade away as also observed in our previous phase IIa trial in Kabul [12]. In Group II, two scar keloïds (8.6%, 4 mM in diameter) formed at D172 and D239.

## Discussion

This phase IIb study was conducted according to the state of the art in the analysis of chronic wound healing [28],[29] and anticipated recommendations for RCTs in CL [30]. Rapid healing in Group II confirmed the robustness of the findings in the Kabul trial [12].

### Limitations

By its nature the present trial could not be conducted as either a double or a single blinded trial due to the physical nature of the applied interventions.

### Generalizability

So far, six physical treatment regimens have been proposed and clinically tested to disinhibit the healing delay of chronic wounds. In CL ulcers, thermotherapy (TT) [13],[31],[32] sparing host tissue, photodynamic therapy (PDT) [33]-[35] producing tissue ^1^O_2_ [36], cryotherapy (N_2_) [37]-[39], CO_2_ laser [40]-[43], and HF-EC [12] all elicited short-term constructive inflammatory reactions with beneficial ROS production in the tissue [44] and destroyed parasites and commensal bacteria together with host cells. Similarly, CAP presumably also acted by forming ROS in non-CL skin lesions [45],[46]. After initial debridement by HF-EC CL ulcers healed despite of residual, persisting parasites, and MWT treatment with pharmaceutical chlorite showed an additional beneficial healing effect in lesions with high pre-treatment parasite loads [12]. In 2008, González et al. highlighted on page 33 of their Cochrane meta-analysis: “We found no RCTs on the use of wound healing to treat OWCL” [47]. This initiated the introduction of MWT with 0.045% DAC N-055 alone as third regimen in the present RCT, without previous HF-EC treatment.

### Interpretation

Pharmaceutical chlorite did not shorten wound-healing times in the Kabul trial [12], when it was performed after HF-EC wound debridement, which already promoted wound granulation to a maximum extent. However, in spite of the small sample size of the present trial, MWT with 0.09% DAC N-055 alone showed a significantly shorter wound closure time in the PP analysis than topical anti-parasitic SSG, suggesting that pharmaceutical chlorite has an intrinsic effect on wound healing, even if such wounds result from parasitic infections.

Reactive oxygen species (ROS) seem to be prominently involved in wound healing pathology and physiology. At tissue levels above 10^-4^ M, ROS including ^1^O_2_ are cytotoxic and may act as cell aging signals [22],[48]; below a tissue concentration of 10^-5^ M, ROS promote cell proliferation and tissue repair (granulation, neo-angiogenesis and epithelialisation) [22]. This explains the dose-related effect of NTP on mammalian cells, which produces ROS [11], ranging from inducing apoptosis to increasing cell proliferation [44]. This could also explain, why non-constructive inflammation with ROS production >10^-4^ M inhibits wound healing, whilst constructive inflammation with ROS production <10^-5^ M promotes tissue regeneration and wound closures [49],[50], similarly to CAP [51].

Liquid nitrogen, the CO_2_ laser, bipolar HF-EC, PDT, and pharmaceutical chlorite applied to the wound [3],[5],[6] or to the skin [17],[18], where NaClO_2_ reacts with heme or bacterial dismutases, are likely to have a common denominator for the of wound healing, which might be ^1^O_2_ or other ROS.

0.045% DAC N-055 basic crème contains 5 mM NaClO_2_ diffusing slowly into the skin and wound tissue. For NaClO_2_ to act as a protracted source of ^1^O_2_ in the range of 10^-5^ to 10^-6^ M in the wound tissue, it would be sufficient that 1 to 10‰ of the applied DAC N-055 inoculum enters a heme-catalyzed dismutase reaction which leads to the formation of hypochlorite and subsequently to ^1^O_2._ In 1984, it was apparently wrong to speculate on pharmaceutical chlorite as a direct source of oxygen supply for the respiratory chain [52].

Skin defects are always at risk of microbial colonization and infection, especially in the problematic hygienic hospital environment of poor countries such as Afghanistan. Therefore, rapid wound closure is highly desirable. Moreover, physical treatment techniques have the advantage that they do not induce parasite resistance, which is an increasing concern for CL therapy with pentavalent antimony [53].

In contrast to HF-EC, less intrusive therapies such as the application of N_2_ or PDT are not administered in “one session only” and require supposedly more resources. Both the CO_2_ laser and bipolar HF-electrosurgery have been used for single session debridement in patients with as many as 4-5 lesions (Reto Steiner and KW Stahl, German Medical Service Kabul, unpublished results). However, in contrast to the CO_2_ laser, the HF-EC device is a robust instrument with practically no maintenance costs for decades. The HF-EC device can be run with a car battery in the absence of electrical power supply. In contrast to a CO_2_ laser, bipolar HF-EC treatment is more superficial, allows a loophole-free destruction of the lesion tissue, and the cauterization process is less unrestricted in space than with laser beams, which induce deep and very narrow thermal tissue coagulation [54]. From our experience in Kabul with the German Medical Service (GMS), the CO_2_ laser seems to be especially helpful to treat multiple small recurrent CL lesions in the face.

Already in 2005, results from murine experiments have claimed that the wound repair response controls the outcome of cutaneous leishmaniasis [55], but so far, to our knowledge, this has not inspired any clinical research work to test this hypothesis in humans. Of course, direct extrapolation from “mice to men” is problematic and our clinical results do not give any straightforward answer to the question, whether mechanisms found in the mouse model also apply to humans [56]-[58]. However, it is noteworthy to mention that a high degree of microbial and fungal contaminations and superinfections have been observed in leishmanial wounds [15]. They lead to chronic inflammatory processes which counteract the wound healing and involve peroxidase secretions from macrophages and neutrophils. Interestingly, recent biochemical work has shown that human peroxidases, such as lactoperoxidase and myeloperoxidase, are key targets of μM concentrations of chlorite, which destroys these enzymes by heme catalysis [59].

### Non-desired effects

Every scarring process of full thickness wounds with or without topical CL treatment comprises an inevitable risk of late keloïd formation. Keloïds were registered in six (5.3%) patients within the phase IIa trial in Kabul [12] and in two (8.7%) patients of Group II in the present RCT. Asilian and colleagues found hypertrophic scars in five (6%) patients treated with CO_2_ laser [41]. Wound healing in the absence of a strong parasitocidal intervention (Group III) seemed to bear a higher risk of reulcerations than observed in Groups I and II, where parasites were killed by SSG or HF-EC, respectively. As DAC N-055 has a strong wound healing effect, but only a limited leishmanicidal activity notably against intracellular parasites [12] (US and CB, unpublished data), future clinical trials should investigate the combination of DAC N-055 MWT with a strong leishmanicidal regimen.

## Conclusions

In CL endemic regions with poor infrastructure, bipolar HF-EC is a robust technology to debride CL lesions under local anaesthesia. The combination with DAC N-055 MWT provides additional anti-parasitic, antimicrobial and wound healing effects. Well designed and controlled prospective cohort studies with a large sample of patients and a post-treatment monitoring period of one to two years are the only way of investigating the frequency and impact of NDEs such as keloïd or hypertrophic scars, permanent pigmental disorders, or persisting erythemas. The cost-effectiveness of the proposed interventions is currently under investigation.

## Authors' contributions

The authors accept full responsibility for the overall content of this report. HCS, KWS, CB, and JLB designed the trial. KWS and IS were the principal investigators for the clinical part of the trial, whereas the CB and US were principal investigators for the laboratory part of the trial; FA, MLA, and IS enrolled and managed patients, collected laboratory and clinical data; HCS, KWS, US, and CB contributed to writing of the paper. HCS and JLB analysed the data. RS participated in supervision. All authors read and approved the final manuscript.

## Additional files

## Electronic supplementary material

Additional file 1: Formula of 0.045% DAC N-055 jelly (adjusted with NaOH to pH 7-8). (XLSX 34 KB)

Additional file 2: Formula of 0.045% DAC N-055 basic cream (adjusted to pH 8 with acetic acid). (XLSX 31 KB)

Additional file 3: **Decision rules in the adaptive drop-loser statistical sample design with α**_**1**_ **=0.01, β**_**1**_ **=0.15 and α**_**2**_ **=0.1871.** (XLSX 9 KB)

Additional file 4: **Patients that could not be considered in the per protocol (PP) statistical analysis.** Three (9.3 %) patients in Group II regimen and three (9.7 %) patients in Group III dropped out during treatment. There were no differences in the dropout rates between the regimens within the ITT analysis (p=0.374, Chi-Square (Exact Sign.)). Eighteen patients listed in Additional file 4, out of the total of 87 randomized patients, could not be considered in the PP analysis due to protocol breaches. Two of these patients with mixed treatment were kept in the ITT analysis after being assigned to their randomized groups. (XLSX 34 KB)

Below are the links to the authors’ original submitted files for images.

Authors’ original file for figure 1

Authors’ original file for figure 2

Authors’ original file for figure 3

Authors’ original file for figure 4

## Abbreviations

AA: German Federal Foreign Office
AFPAK: Afghanistan and Pakistan Stabilization Pact
BMBF: German Federal Ministry for Education and Research
BMZ: German Federal Ministry for Economic Cooperation and Development
CAP: Cold atmospheric plasma
CL: Cutaneous leishmaniasis
DAAD: German Academic Exchange Service
DAC: German Drug Codex
DAC N-055: Pharmaceutical sodium chlorite listed in the German Drug Codex
EC 1.11.1.6: Enzyme classification
EC: Bipolar electrocauterization
GMS: German Medical Service
HF: High-frequency
ITT: Intention to treat
IZKF: Interdisciplinary Centre of Clinical Research
*L. major*: *Leishmania major*
*L. tropica*: *Leishmania tropica*
LMC: Leishmania and Malaria Centre
MoPH: Ministry of Public Health
MWT: Moist wound treatment
NDE: Non desired effects
NMLCP: National Malaria and Leishmania Control Program
NTP: Non thermal plasma
PP: Per protocol
RCT: Randomized controlled trial
ROS: Reactive oxygen species
SSG: Sodium stibogluconate
WHO: World Health Organization
