# Supplementary figures and images for: A randomized controlled phase IIb wound healing trial of cutaneous leishmaniasis ulcers with 0.045% pharmaceutical chlorite (DAC N-055) with and without bipolar high frequency electro-cauterization versus intralesional antimony in Afghanistan
Source: BMC Infect Dis. 2014 Nov 25;14:619. doi: 10.1186/s12879-014-0619-8 (PMC4258014; doi:10.1186/s12879-014-0619-8)

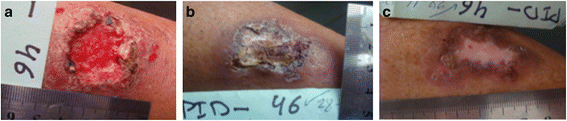

Supplement: Supplementary file 5 — Authors’ original file for figure 1 [file 12879_2014_619_MOESM5_ESM.gif]

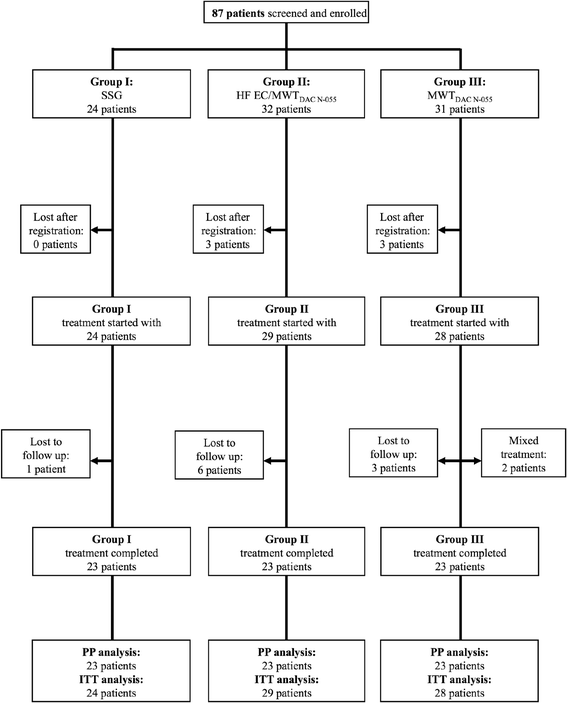

Supplement: Supplementary file 6 — Authors’ original file for figure 2 [file 12879_2014_619_MOESM6_ESM.gif]

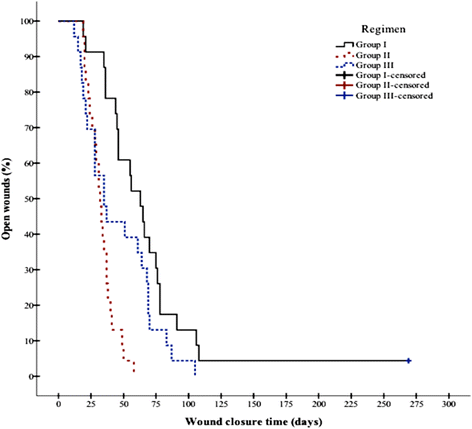

Supplement: Supplementary file 7 — Authors’ original file for figure 3 [file 12879_2014_619_MOESM7_ESM.gif]

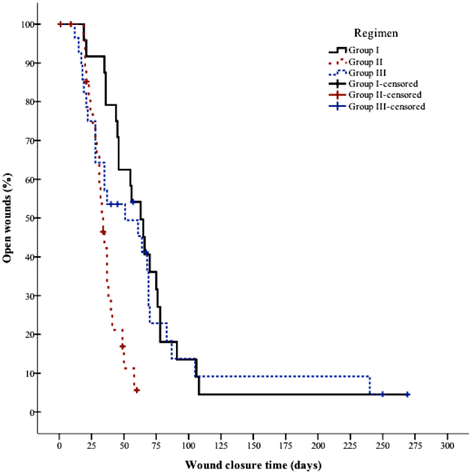

Supplement: Supplementary file 8 — Authors’ original file for figure 4 [file 12879_2014_619_MOESM8_ESM.gif]
